# The parabrachial-to-amygdala pathway provides aversive information to induce avoidance behavior in mice

**DOI:** 10.1186/s13041-021-00807-5

**Published:** 2021-06-24

**Authors:** Mariko Ito, Masashi Nagase, Suguru Tohyama, Kaori Mikami, Fusao Kato, Ayako M. Watabe

**Affiliations:** 1 Department of Neuroscience, The Jikei University School of Medicine, Tokyo, Japan; 2 Institute of Clinical Medicine and Research, Research Center for Medical Sciences, The Jikei University School of Medicine, 163-1 Kashiwa-shita, Kashiwa, Chiba 277-8567 Japan; 3 Department of Anesthesiology, The Jikei University School of Medicine, Tokyo, Japan

**Keywords:** Aversive information, Pain, Central amygdala, Optogenetics, Mouse, Defensive behavior

## Abstract

The neuronal circuitry for pain signals has been intensively studied for decades. The external lateral parabrachial nucleus (PB) was shown to play a crucial role in nociceptive information processing. Previous work, including ours, has demonstrated that stimulating the neuronal pathway from the PB to the central region of the amygdala (CeA) can substitute for an actual pain signal to drive an associative form of threat/fear memory formation. However, it is still unknown whether activation of the PB–CeA pathway can directly drive avoidance behavior, escape behavior, or only acts as strategic freezing behavior for later memory retrieval. To directly address this issue, we have developed a real-time Y-maze conditioning behavioral paradigm to examine avoidance behavior induced by optogenetic stimulation of the PB–CeA pathway. In this current study, we have demonstrated that the PB–CeA pathway carries aversive information that can directly trigger avoidance behavior and thereby serve as an alarm signal to induce adaptive behaviors for later decision-making.

## Introduction

Detecting and avoiding potential harm is crucial for the survival of animals. Aversive stimuli therefore potently induce defensive behaviors and trigger fear memory formation. Elucidating the neuronal circuitry mechanisms underlying these defensive behaviors has been a fundamental question in the research field of fear and anxiety [1–5].

The amygdala is one of the brain regions extensively studied for the encoding and processing of negative valence of sensory signals [1, 3, 6–9]. Plasticity mechanisms of fear/threat conditioning [10], in which aversive valence of nociceptive signals such as electric shock are associated with previously neutral sensory information such as tone or context, have been intensively studied in recent decades. However, there is only limited evidence on how the aversive valence of the nociceptive signal itself is regulated in the amygdala [11]. One of the subdivisions of the amygdala, the central amygdala (CeA), has been shown to integrate various sensory information and orchestrate appropriate defensive behaviors such as fight or flight [12]. The CeA receives nociceptive information indirectly from other subdivisions of the amygdala such as the basolateral amygdala and/or lateral amygdala via a well-characterized conventional spinal–thalamic pathway [6, 13], as well as directly from the pontine parabrachial nucleus (PB) [14–16]. Indeed, the lateral PB is well characterized as a predominant target of ascending nociceptive-specific neurons in the spinal dorsal horn, as part of the spino–parabrachio–amygdaloid pathway [17–21]. Previous studies have demonstrated that nociceptive stimuli increase neuronal activity as well as c-Fos immunoreactivity in the PB [22, 23]. Also, recent studies showed that activation of the spino-parabrachial pathway evokes pain-related behaviors and aversive responses [20, 21], while activation of the excitatory neurons in the PB induces neuropathic pain-like behaviors [24]. Furthermore, the PB nociceptive neurons have been shown to receive monosynaptic inputs from craniofacial sensory neurons, as part of the trigemino-parabrachio pathway [25].

These anatomical and physiological lines of evidence suggest that the PB may be involved in the emotional aspect of nociceptive information transmission into the CeA. In support of this, we have previously demonstrated that without providing any physical pain stimuli to mice, optogenetic activation of the presynaptic terminals of PB projection neurons into the CeA serves as an unconditioned stimulus (US) in fear/threat conditioning, suggesting that the PB–CeA pathway encodes an emotional aspect of pain information of the US, which drives the associative form of fear learning [26]. Furthermore, a specific subpopulation of the PB that expresses calcitonin gene-related peptide (CGRP) neurons has been demonstrated to play a key role in the termination of food intake [27], fear memory formation [28], pain memory recall [29], and conditioned taste aversion [30]. A recent study from the Ross group also demonstrated that optogenetic stimulation of the PB–CeA pathway resulted in aversive learning [31].

Although these lines of evidence suggest that the PB–CeA pathway is involved in aversive pain information, it is still unclear whether this pathway can directly drive avoidance behavior, escape behavior, or only acts as strategic freezing behavior for later memory retrieval. To directly address this question here, we have developed a real-time Y-maze conditioning behavioral paradigm to access avoidance behavior induced by optogenetic stimulation of the PB–CeA pathway in a time- and experience-dependent manner.

## Materials and methods

### Animals

All the experimental protocols in this study including the use of animals were approved by the Institutional Animal Care and Use Committee of The Jikei University (Tokyo, Japan) (Approval Nos. 21–061, 2016–032). All experiments complied with the *Guidelines for Proper Conduct of Animal Experiments* by the Science Council of Japan (2006) and those recommended by the International Association for the Study of Pain. All efforts were made to reduce the number of animals used and the suffering of the animals. Male C57BL/6 J mice (CLEA Japan Inc., Tokyo, Japan) were group-housed and provided with food and water ad libitum on a 12 h light/dark cycle.

### Surgical procedures for viral vector microinjection

Four-week-old male C57BL/6 mice were used for viral injections with an adeno-associated virus (AAV5) encoding channelrhodopsin (ChR2) fused to YFP under control of the synapsin promoter (AAV5-hSyn-ChR2(H134R)-eYFP (University of Pennsylvania Vector Core, Philadelphia, PA, USA) or a control virus carrying only GFP (AAV5-hSyn-eGFP; University of Pennsylvania Vector Core). Mice were intraperitoneally anesthetized with pentobarbital sodium (45–50 mg/kg) and fixed in a stereotaxic device. An additional 0.5% isoflurane was administered via a nasal mask throughout the surgery to obtain the appropriate depth of anesthesia. The AAV (0.25 μl) was microinjected bilaterally into the external lateral PB (6.2 mm posterior to bregma, 1.5 mm lateral to midline, and 4.4 mm ventral to the cortical surface, with a 20° anterior-to-posterior angle to avoid damaging the superficial arteries during surgery) using a Hamilton microsyringe (1701RN Neuros Syringe, 33 G, 10 μl; Hamilton Company, Reno, NV, USA), as previously described [26]. The injection speed (50 nl/min) was controlled by a micro syringe pump (UltraMicroPumpII with SYS-Micro4 Controller, UMP2, UMC4; World Precision Instruments, Sarasota, FL, USA). Injection syringes were left in place for 10 min before withdrawing.

Five weeks later, a second surgical procedure was performed for the placement of a bilateral light-emitting diode (LED) cannula unit consisting of dual optical fibers (0.25 mm in diameter, 4.0 mm in length, and 5.0 mm in spacing) attached to a LED body (blue, 470 nm) (TeleLCD-B-4–250-5; Bio Research Center, Tokyo, Japan). Mice were allowed to recover for 7–8 days. The LED cannula unit was stereotactically inserted to target the CeA (1.4 mm posterior to bregma, 2.5 mm lateral to midline), and fixed to the skull with dental cement (GC Fuji I; GC Corporation, Tokyo, Japan).

### Real-time Y-maze conditioning paradigm

**Fig. 1:**
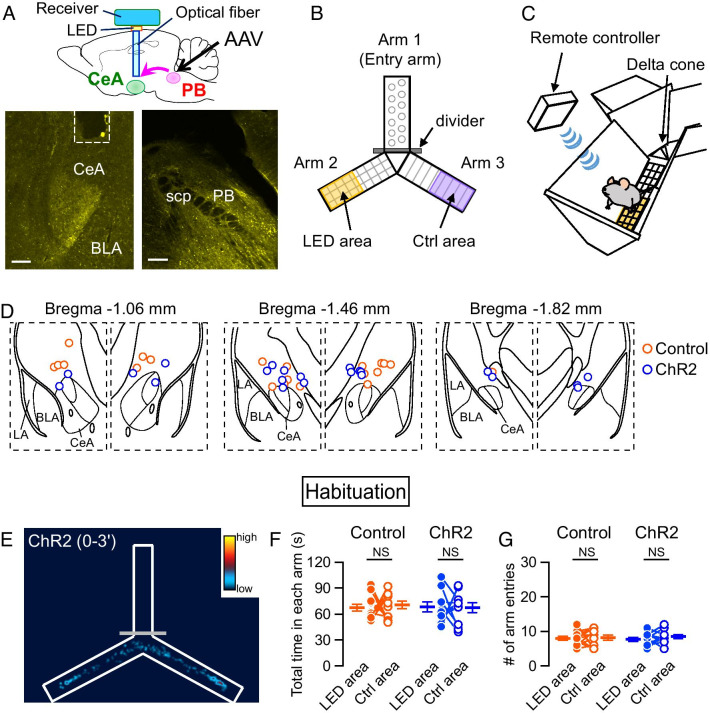
Experimental paradigm of Y-maze apparatus.** A** Schematic of the experimental configuration (top) and representative images of YFP fluorescence in the CeA, projection site (bottom left), and the PB, injection site (bottom right). The dashed line in the bottom left image indicates the position of the optic fiber. Scale bar, 150 μm. **B**, **C** Schematic of the Y-maze apparatus and photostimulation area (**B**) and wireless LED stimulation system (**C**). **D** Schematic illustrations of the CeA and the position of the LED cannula tips for photostimulations. Each circle represents the approximate cannula tip placements (orange, control mice; bule, ChR2 mice). The tip placements of one mouse in the control group was not plotted because some damages in the brain slices made it difficult to estimate the tip positions. **E** Heatmap showing the time spent in the arms for a mouse during habituation. **F**, **G** Summary of the total time spent in each arm (**F**) and the number of arm entries (**G**). Circles indicate each mouse. Control, *n* = 11; ChR2, *n* = 10. NS, not significantly different (Wilcoxon signed rank test)

All behavioral training and testing were conducted in a custom-built Y-shaped maze apparatus (YM-3002; O’Hara & Co., Ltd., Tokyo, Japan) placed in a sound-attenuating chamber (CL-M3; O’Hara & Co., Ltd.) (Fig. 1A, B). The Y-maze consists of three arms (3 cm wide on the bottom and 12 cm wide on the top, length 16 cm, height 12 cm) with a 120° angle between the two arms. The sidewalls of the arms were angled, and a delta cone was placed in the center triangle area to promote the mouse to move in the arms. The floor of each arm had a different pattern-designed metal plate: Arm 1 was made of punched metal, Arm 2 of grid metal, and Arm 3 of mesh metal.

Five days before conditioning, mice were habituated daily to dummy Teleopto-receivers (2 g, TeleDummy; Bio Research Center) attached to their heads and handling for 5 min. One day before conditioning, mice were habituated for 3 min in the Y-maze apparatus (50 lx, 50 dB background white noise) with dummy Teleopto-receivers. At the beginning of each habituation session, the mouse was placed into Arm 1 and as soon as the mouse entered the central triangular area, a divider was inserted so that the mouse could only freely move between the Arm 2 and Arm 3 areas. The sound-attenuating chamber door was immediately closed, and mouse behavior was monitored for 3 min.

On the conditioning day, all mice were transferred from their home cage to the waiting room and acclimated for 60 min before the start of conditioning. During the acclimation period, each mouse was attached to the dummy receiver, which was then replaced by a bilateral LED cannula unit of the Teleopto-receiver (TeleR-2-P, an infrared-driven wireless LED unit; Bio Research Center) immediately before the conditioning session. The conditioning session was conducted in a similar manner as the habituation session, and mouse behavior was monitored for 10 min. The LED stimulating program was designed by the Time OFCR1 software (O’Hara & Co., Ltd.); specifically, whenever the mouse entered the LED area in Arm 2 (shown as a yellow rectangle in Fig. 1B), it received LED illumination (5 ms, 40 Hz, 4.5 mW) that was controlled by an infrared-driven remote controller (Teleopto remote controller; Bio Research Center) located on the inner wall of the sound-attenuating chamber (O’Hara & Co., Ltd.). A programmable stimulator (Master-8; A.M.P. Instruments Ltd., Jerusalem, Israel) and Time OFCR1 software (O’Hara & Co., Ltd.) allowed the delivery of light pulses to be precisely controlled.

The next day, a retrieval test was conducted. Mice were transferred from their home cage to the waiting room and acclimated for 60 min, as on the conditioning day. The retrieval session was conducted in the same manner as the conditioning session in the Y-maze apparatus, except that the mouse was attached to a dummy LED receiver throughout the session, thereby no LED illumination was applied, and behavioral activity was recorded for 5 min. Mouse behavior was captured using a digital camera at 2 frames/s. Time spent in each arm, the LED area, and the control (Ctrl) area, and the number of each arm entries were analyzed using Time OFCR1 software (O’Hara & Co., Ltd.).

### Histology

After all the behavioral experiments, mice were anesthetized with isoflurane (5%) and sacrificed for histological analysis. The brains were extracted and post-fixed in 4% paraformaldehyde overnight at 4 °C, and then placed in 20% sucrose. The brain blocks were frozen after embedding into freezing compound (O.C.T. Compound, Sakura Finetek Japan, Tokyo, Japan), and frozen specimens were sectioned on a cryostat (CM 1850; Leica Microsystems, Wetzlar, Germany). The sections including PB were at a thickness of 40 μm for verification of transfection in PB neurons. The sections including CeA were at a thickness of 25 μm for identification of the position of the LED cannula. Some brain blocks containing PB were embedded in 1.6% low melting point agarose surrounded by 5% agar in cold phosphate-based buffer (pH 7.4) and sectioned transversely into 100-μm-thick sections with a vibrating tissue slicer (PRP7; Dosaka EM, Kyoto, Japan). The sections were mounted on glass slides to obtain images of ChR2-YFP and GFP using a fluorescent microscope (BX63; Olympus, Tokyo, Japan).

### Data analysis

Values are expressed as mean values ± SEM. Differences in values were analyzed using the Wilcoxon signed rank test and Mann–Whitney *U* test. *P*-values for multiple comparisons were corrected with the false discovery rate (Benjamini–Hochberg method). Differences were considered statistically significant at *P* < 0.05.

## Results

### Y-maze conditioning paradigm

To determine whether the PB–CeA pathway governs avoidance behavior or other behaviors, we examined real-time behavior of freely moving mice by combining a Y-maze apparatus with optogenetic activation of this pathway. We bilaterally injected AAV vector carrying ChR2 (hSyn-ChR2(H134R)-eYFP) or control (hSyn-eGFP) into the PB, and implanted dual optic cannulae over the CeA in a similar manner as we previously reported (Fig. 1A) [26]. While it is possible that stimulation of the CeA with this configuration may result in antidromic activation and ectopic stimulation of other PB target regions, recent studies have demonstrated it is not highly likely [31, 32]. As shown in Fig. 1B, the experimental apparatus was Y-shaped with the three arms composed of different textures. The sidewalls of the arms were angled, and a delta cone was placed in the center triangle area, to promote the mouse to move in the arms. A LED illumination area (LED area) was set on the distal half of Arm 2. During the conditioning session, optical stimuli were applied with a wireless system whenever the mouse entered the LED area, and sustained until the mouse exited the LED area (Fig. 1B, C). For comparison, a non-illuminated control area (Ctrl area) was set on the opposite side in Arm 3 as the exact same size as the LED area (Fig. 1B). Placement of the LED cannula was confirmed after all the behavioral experiments were conducted, and they are indicated as circles representing the approximate cannula tip placements in the schematic illustrations of the CeA (Fig. 1D).

One day before conditioning, mice were habituated to the Y-maze apparatus with dummy Teleopto-receivers attached to their heads. At the beginning of each habituation session, the mouse was placed into Arm 1 and as soon as the mouse entered the central triangular area, a divider was inserted so that the mouse could only freely move between the Arm 2 and Arm 3 areas. Mouse behavior was monitored for 3 min, and the time spent in the area during the habituation session was shown as a pseudo-color plot (Fig. 1E). There were no significant differences in total time spent in each arm and number of entries into the arms between the control and ChR2 groups (Fig. 1F, G), suggesting that mice have no bias in texture or right–left preference in our experimental conditions.

### Real-time aversion test using the Y-maze apparatus

**Fig. 2:**
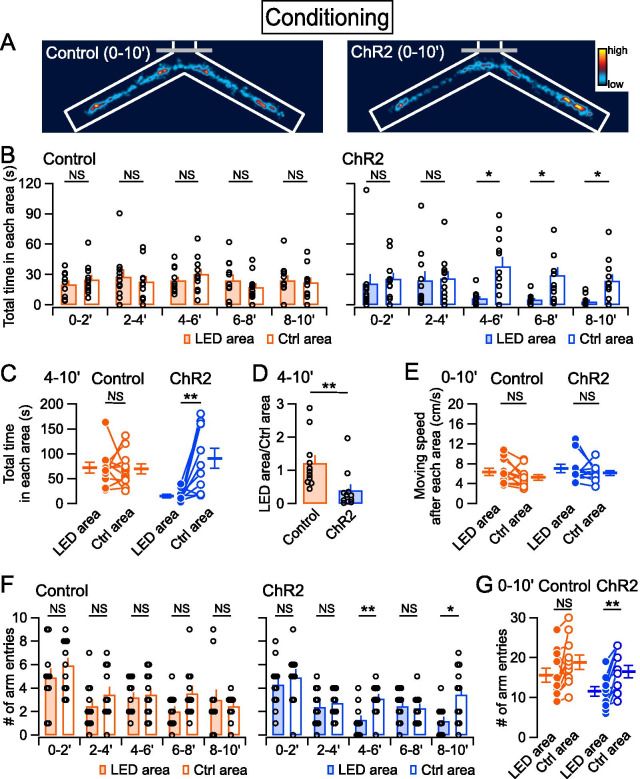
Effects of photoactivation of the PB–CeA pathway on avoidance behavior during conditioning.** A** Heatmaps showing the time spent in the arms during conditioning sessions in the control (left) and ChR2 (right) groups. **B** Average total time spent in the LED (filled bars) and no-LED (open bars) areas every 2 min during a conditioning session in the control (orange) and ChR2 groups (blue). **C** Summary of total time spent in each area during the 4- to 10-min period of a conditioning session. **D** Summary of the ratio of time spent in the LED area to no-LED area during the 4- to 10-min period. **E** Summary of moving speed after leaving each area; this was calculated from the distance traveled for one frame immediately after leaving each area. **F** Average number of entries from the delta cone area to each arm every 2 min in a conditioning session. **G** Summary of the total number of arm entries during a whole conditioning session. Control, *n* = 11; ChR2, *n* = 11. *P < 0.05; **P < 0.01; ***P < 0.001; *NS* not significantly different (Wilcoxon signed rank test in **B**, **C**, **E**, **F** and **G**; Mann–Whitney *U* test in **D**). *P*-values were corrected with the false discovery rate in panels **B** and **F**

Twenty-four hours after habituation, a conditioning session was performed for 10 min. Mice were attached to the bilateral LED cannula unit of the Teleopto-receiver on their heads, and received photostimuli (5 ms, 40 Hz) whenever they entered the LED area, as shown in Fig. 1B. Although ChR2-expressing mice explored both areas during the early phase of the conditioning session (0–4 min), they gradually began to avoid the LED area from 4 min after the start of the conditioning session (Fig. 2A, B). As a result, the total time spent in the LED area during the 4- to 10-min period was significantly shorter compared with the Ctrl area (Fig. 2C). By contrast, the control mice, which received the same photostimuli but did not express ChR2, showed almost the same time spent in both areas throughout the whole conditioning session (Fig. 2A–C), suggesting that activation of the PB–CeA pathway leads to avoidance behavior in a time-dependent manner. To examine the behavior of each mouse, we calculated the ratio of time spent in the LED area over that in the Ctrl area during the 4- to 10-min period. In the ChR2 group, the ratio was significantly lower compared with controls (Fig. 2D), supporting the notion that the PB–CeA pathway serves as an aversive signal driving avoidance behavior during the conditioning session. The distance traveled immediately after leaving the LED area and the Ctrl area, which represents the speed moving out of the area, was not significantly different between the ChR2 and control groups (Fig. 2E), suggesting that the PB-CeA pathway is not involved in escaping behaviors. We also analyzed the number of entries into Arm 2 and Arm 3, which may represent a behavioral choice more strongly than the time spent in each area. The number of entries into Arm 2 (the LED arm), tended to gradually decrease in the ChR2 group, but not the control group (Fig. 2F). The total number of Arm 2 entries in the conditioning session was significantly lower compared with Arm 3 entries in the ChR2 group (Fig. 2G), suggesting that activation of the PB–CeA pathway also alters a behavioral choice. Altogether, these results suggest that the PB–CeA pathway transmits aversive information that causes real-time avoidance behavior.

### Retrieval test with Y-maze conditioning

**Fig. 3:**
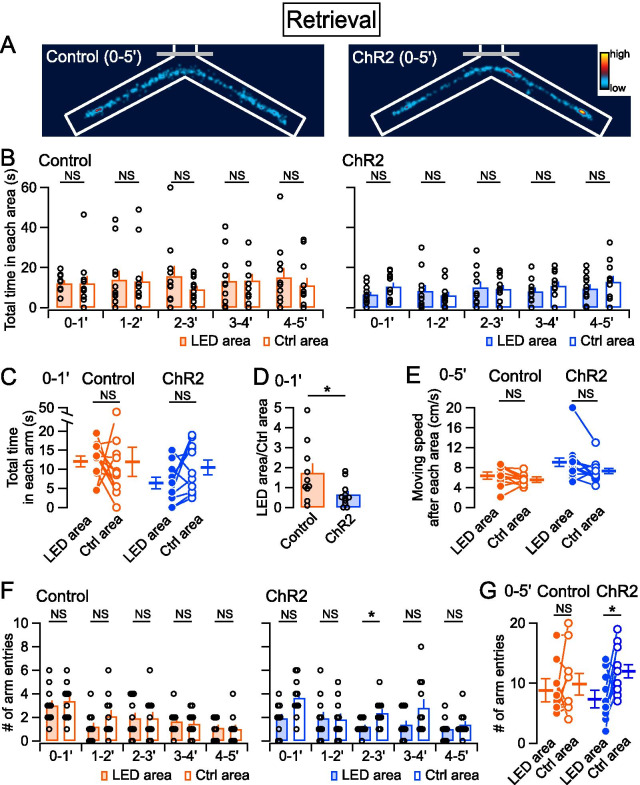
Summary of retrieval behavior.** A** Heatmaps showing the time spent in the arms during a retrieval session in the control (left) and ChR2 (right) groups. **B** Average total time spent in the light-emitting diode (LED) (filled bars) and no-LED (open bars) areas every 1 min during a retrieval session in the control (orange) and ChR2 groups (blue). **C** Summary of total time spent in each area during the first minute of a retrieval session. **D** Summary of the ratio of time spent in the LED area to no-LED area during the first minute. **E** Summary of moving speed after leaving each area. **F** Average number of arm entries every 1 min during a retrieval session. **G** Summary of the total number of arm entries during a whole retrieval session. Control, *n* = 11 (*n* = 10 in **D**, because the ratio could not be calculated in a mouse that did not enter the LED area at the first minute); ChR2, *n* = 11. *P < 0.05; **P < 0.01; NS, not significantly different (Wilcoxon signed rank test in **B**, **C**, **E**, **F** and **G**; Mann–Whitney *U* test in). *P*-values were corrected with the false discovery rate in **B** and **F**

Our and other previous reports have shown that activation of the PB–CeA pathway associated with conditioned stimuli results in aversive memory formation [26, 28, 31]. We therefore performed a retrieval test 24 h later, in which the mice were attached to dummy Teleopto-receivers on bilateral LED cannula units and placed in the same Y-maze apparatus as for the conditioning session. Although some mice tended to avoid the LED area, there were no significant differences in total time spent between the LED area and the Ctrl area in control and ChR2 groups during retrieval sessions (Fig. 3A–C). However, examination of the behavior of each mouse showed that the ratio of total time spent in the LED area over that of the Ctrl area during the first minute was significantly lower in the ChR2 group compared with the control group (Fig. 3D), suggesting that ChR2-expressing mice have formed aversive memory. The distance traveled immediately after the areas were not significantly different (Fig. 3E). These results suggest that the conditioning paradigm used in this study may have established only a weak aversive memory. To analyze their behavior more in detail, we examined the number of arm entries. The number of entries into Arm 2 was significantly less compared with entries into Arm 3 in the ChR2 group at several time points (Fig. 3F). Over the whole retrieval session, there was a significant difference in the number of arm entries to Arm 2 and Arm 3 in the ChR2 group (Fig. 3G). These results suggest that decision-making to select which arm to enter was affected by photoactivation of the PB–CeA pathway on the conditioning day.

## Discussion

In the present study, we found that optogenetic activation of the PB–CeA pathway gradually and potently induced avoidance behavior in a time- and experience-dependent manner using the Y-maze paradigm. These results suggest that the PB–CeA pathway can trigger avoidance behavior and thereby serve as an alarm signal to induce adaptive behaviors later. We have previously reported that optogenetic activation of the PB–CeA pathway serves as the US to substitute actual pain stimulation, resulting in artificial fear memory formation [26]. Collectively, these lines of evidence support the notion that the PB–CeA pathway carries aversive information that can represent the emotional aspect of the pain and drive adaptive behaviors. This is in accordance with the previously suggested role of the PB (especially CGRP-expressing neurons in the PB), in serving as a general alarm system to coordinate defensive behaviors in response to existing and/or potential threats [33, 34].

Recently, the Ross group has skillfully teased apart distinct downstream projections from the PB, finding that cells from the external lateral PB projected to CeA and the bed nucleus of the stria terminalis, while cells from dorsal PB projected to the ventral medial hypothalamus and lateral periaqueductal gray [31]. They also demonstrated that optogenetic activation of axonal terminals of the external lateral PB–CeA pathway (20 Hz/5 ms) induced real-time place avoidance using a two-compartment test, which resulted in aversive learning. Our present results using a 40 Hz/5 ms stimulation paradigm are in good agreement with their analysis. Another recent report from the Palmiter group also demonstrated that photostimulation (15 Hz/10 ms) of CGRP-positive neuronal terminals from the PB to rostral CeA (but not caudal CeA) induced real-time place aversion as well [32]. While it is possible that stimulation of presynaptic terminals of the CeA may result in antidromic activation and ectopic stimulation of other PB target regions, the above two studies have demonstrated it is not highly likely because stimulating presynaptic terminals of different target regions resulted in different behavioral phenotypes.

The Y-maze apparatus in the present study was designed to provide photostimulation only when the animal enters the LED area, which was located in the distal half of Arm 2 in the Y-maze apparatus (Fig. 1B). This configuration allows the animals to freely explore the entire platform at the beginning, allowing threat learning to occur gradually. Consequently, the development of avoidance behavior to the target region was successfully detected at around 4 min after the start of the session, and the behavior gradually developed with time (Fig. 2A, B). Moving speed from the LED area showed no significant difference (Fig. 2E), suggesting that PB–CeA photostimulation does not induce escaping behavior per se. This is consistent with previous demonstration that the PB projections to other brain regions such as the dorsal reticular formation, the ventral medial hypothalamus, and lateral periaqueductal gray, are involved in escaping behavior regulation [31, 35]. Altogether, these results support a notion that the PB-CeA projection plays a crucial role in avoidance behavior.

Nakajima and Minokoshi’s group have previously reported that another subset of neurons in the PB (which express a transcription factor, SATB Homeobox 2 [SatB2]) encodes positive valence of sweet taste signals [36]. They showed that most SatB2-positive PB neurons are responsive to sweet taste but not bitter taste. Furthermore, using a SatB2-Cre mouse line, they found that photostimulation of the projection pathway to the ventral posteromedial nucleus of the thalamus (VPMpc) induces appetitive licking behavior as well as place preference. In contrast to the VPMpc projection pathway, the CeA projection pathway from SatB2-expressing neurons in the PB showed no apparent place preference nor aversion [36]. These results may seem to contradict our present findings, but the expression patterns of SatB2-positive neurons and CGRP-positive neurons are topographically distinct. SatB2 neurons reside in the waist subregion, while CGRP neurons reside in the external lateral subregion. Therefore, different subregions and/or cell-types in the PB may differentially encode appetitive and aversive valence of sensory information.

Interestingly, optogenetic activation of the PB–CeA pathway in the present study did not induce apparent conditioned place avoidance in the Y-maze apparatus on the retrieval day when measured as total time spent in the target area (Fig. 3A, B); meanwhile, the same mice exhibited robust avoidance behavior on the conditioning day (Fig. 2A, B). This may contradict not only the recent study by Chiang et al. [31] but also our previous report showing that PB–CeA photostimulation paired with tone induced cued fear/threat conditioning, so that mice showed significant freezing on the retrieval test [26]. Regarding the LED stimulation pattern between these studies, the experimental conditions are similar, and both used wireless LED stimulation systems of 40 Hz/5 ms. Whereas the fear/threat conditioning used 2 s of LED stimulation paired with 20 s tone co-terminated [26], the present study used LED stimulation that was only administered to the mice when they entered the LED area. Therefore, the most straightforward explanation for this apparent difference is that mice that experienced more “aversive” signals by entering the target area will more readily avoid the area and thereby receive less photostimulation on the conditioning day. Thus, less associative learning will have formed between the stimulation and context. However, this possibility is not highly likely because when we analyzed the number of arm entries from the central triangular area, the mice exhibited significantly fewer entries to the stimulated arm on retrieval tests (Fig. 3G). The number of arm entries is measured when animals are located in the central triangular area and enter either Arm 2 or Arm 3, thus reflecting a decision-making process. Collectively, these results suggest that the PB–CeA serves as an aversive signal in the present Y-maze conditioning paradigm, in which freely walking mice are conditioned to associate the Arm 2 context with aversive valence, thereby influencing later decision-making for the arm entry from the delta cone area.

The PB converges not only nociceptive information, but also various other sensory information including taste, itch, and thermal information, and then projects to a wide range of brain regions [33]. It is crucial to employ cell type- and target-specific intersectional methods to study how they are involved in processing diverse sensory modalities and their physiological consequences.

## Data Availability

All data are available upon request to the corresponding author.

## Abbreviations

AAV: Adeno-associated virus
CeA: Central region of the amygdala
ChR: Channelrhodopsin
LED: Light-emitting diode
PB: Parabrachial nuclei
US: Unconditioned stimulus
